# Natural selfish genetic elements should not be defined as gene drives

**DOI:** 10.1073/pnas.2201142119

**Published:** 2022-08-15

**Authors:** Mark A. Wells, Ricarda A. Steinbrecher

**Affiliations:** ^a^EcoNexus, Oxford OX4 9BS, United Kingdom

Gene drives are increasingly discussed in the political realm, and how the term is defined therefore has important implications. The opinion piece from Alphey et al. (1) identifies a lack of consensus on the definition and makes explicit changes in how the terminology is being used by some researchers. As such it is a timely invitation for debate.

The definition of the term “gene drive” Alphey et al. (1) propose would include naturally occurring selfish genetic elements (SGEs) and natural processes causing biased inheritance. We disagree with this aspect of the proposal, which does not reflect the original use of the term, which related to engineered systems. The altered definition has the effect of emphasizing the similarity of engineered gene drives to natural SGEs by allowing both to be described as “gene drives.” The use of this technology brings serious risks (2, 3)—we therefore are concerned that the proposed terminology presents engineered gene drive systems as repurposed natural entities. This unfortunately brings connotations of safety and familiarity that will discourage the necessary scrutiny. We thus regard the proposed definition as problematic in the context of political and regulatory discussions concerning this technology. For example, we find it worrying that an International Union for Conservation of Nature report, which will be influential in the regulatory debate in the conservation community, introduced “gene drive” as “a ubiquitous natural phenomenon” (4).

There are significant differences between the engineered and natural systems which play out at the level of risks. Engineered gene drive systems would evidently be used to serve human intentions. While these intentions themselves deserve scrutiny, they will also fundamentally affect the composition, properties, and genomic context of the resulting engineered elements. Interaction of these elements with evolutionary forces may further influence their design: Consider homing gene drives that thwart the emergence of resistance (5). Many engineered gene drive designs would also result in the permanent incorporation of homing endonuclease genes into genomes of multicellular eukaryotes, which has no known precedent in nature (6). In the context of the regulatory debate it is appropriate to use terminology that draws attention to such differences.

Proposals to artificially “drive” genes into wild insect populations (e.g., ref. 7) predate the term “gene drive,” which began to appear just over fifteen years ago (e.g., refs. 8–10). The term was originally used to describe technological approaches, including use of engineered SGEs, to force novel genes into wild populations to purposefully modify, suppress, or eliminate such populations. An expanded meaning of the term “gene drive” that encompasses natural phenomena and natural SGEs has been introduced by some in the research community over recent years, without the rationale ever being made explicit. Alphey et al. (1) argue to formalize these changes. Given the terminology has ramifications for the broader regulatory and political discussions, these changes require critical analysis and debate, including by parties outside the gene drive research community. In our view a narrower definition, with intentionality and use of genetic engineering as defining features, is more appropriate, especially in the context of risk assessment.
